# Co-Designing the Primary Care Individual Social Norms Musculoskeletal Data Dashboard (PRISM) for First Contact Physiotherapy: Consensus-Based Qualitative Study

**DOI:** 10.2196/105148

**Published:** 2026-10-20

**Authors:** Emma Dunphy, Alexander Braybrooke, Jamie Ross, Irwin Nazareth, Fiona Stevenson

**Affiliations:** 1 Department of Primary Care and Population Health, University College London, Upper third floor, Royal Free Medical School, London, England, NW3 2PF, United Kingdom, 1 07510146528; 2 School of Medicine, Keele University, Staffordshire, England, ST5 5BG, United Kingdom; 3 Centre for Primary Care, Wolfson Institute of Population Health, Barts and The London School of Medicine and Dentistry, The Wolfson Institute, Queen Mary University of London, London, England, United Kingdom

**Keywords:** first contact physiotherapy, primary health care, quality indicators, cooperative behavior, social norms, musculoskeletal diseases, clinical supervision, qualitative research, health care

## Abstract

**Background:**

In the United Kingdom, first contact practitioners (FCPs) provide direct access to musculoskeletal (MSK) assessment and management in primary care. This means FCPs have to assess patients with no prior medical screening, which assumes reliance on developed skills and experience. The pool of appropriately experienced physiotherapists is limited, and less experienced clinicians have been recruited. A 2026 national evaluation found substantial variation in FCP service delivery, with data collection and governance among its structural causes. The Primary Care Individual Social Norms MSK Data Dashboard (PRISM) dashboard gives FCPs feedback on their clinical activity relative to their peers to support reflection toward improving MSK care.

**Objective:**

This study aimed to co-design the metrics, filters, and guidance content of the PRISM dashboard and guidebook to reflect evidence-based practice and the real-world context of FCP roles, and apply a critical realist approach to identify the mechanisms and conditions shaping its design.

**Methods:**

This qualitative co-design study used a modified nominal group process across 2 online workshops in June and August 2025, with 10 purposively sampled participants: clinical FCPs, national physiotherapy and data leaders, and a patient representative. Two analytic strands were applied to the same data. The consensus process produced the metric set and filters. Critical realist thematic analysis of the transcripts using NVivo (version 12 Plus; QSR International), with line-by-line coding using inductive and a priori codes, identified the conditions shaping how those metrics would need to be interpreted.

**Results:**

Participants reached consensus on a core metric set describing the breadth of the FCP role, separating variation generated by clinical decisions from variation generated by structure such as local diagnostic access, prescribing rights, and referral pathways. Contextual filters were co-designed at patient, clinician, and service level to keep structure visible. Supervision emerged as a condition surrounding the dashboard rather than a component, since the same comparison prompts reflection where role clarity and psychological safety are present and defensiveness where they are not. A tool dependent on supervision would work least well where support is weakest. This means that an option for stand-alone use is a design requirement. Three data integrity risks were identified: recording reliability, data gaming, and clinician burden. The guidebook enables these conditions to be transferred into practice, with separate guidance for FCPs and supervisors and a 4-step reflection structure.

**Conclusions:**

Expert co-design produced a metric set describing the breadth of the role while accommodating variation between services and acknowledging the conditions under which PRISM can function as intended. This paper moves from the national position that FCP services collect data to a specification of the conditions under which collecting data produces learning. Mapping of the metrics against National Institute for Health and Care Excellence MSK guidelines and quality indicators is reported in a companion paper.

## Introduction

In the United Kingdom, first contact practitioners (FCPs) are embedded within primary care settings to provide direct access to musculoskeletal (MSK) assessment and management, reducing demand on general practitioners (GPs) and improving patient access to timely specialist care [1-3]. FCP services were found to provide an opportunity to free up capacity of primary care doctors, and FCPs were best placed to meet the needs of the MSK patients [1,3,4]. Recent national clinical trial evaluation of FCP found that FCPs were as effective and more cost-effective with better MSK outcomes than GPs at certain timepoints in care [5,6].

In recognition of the clinical complexity involved in managing first contact patients without prior medical screening, FCPs are expected to practice at an advanced level in the clinical pillar of practice, consistent with guidance from the Chartered Society of Physiotherapy and Health Education England [7-9]. However, the pool of appropriately experienced physiotherapists is limited, with evidence suggesting that less experienced FCPs are being recruited into these posts, leading to reports of high turnover, increased clinical risk, and unwarranted variation in care [10,11].

Recent research has also shown inconsistent governance, inconsistent scope of practice, isolation, the absence of informal work support, and high workloads [6,10]. Stress, burnout, high turnover, and difficulty holding clinical risk are also commonly reported [10,12-14]. Such challenges mean that it is essential to clearly identify opportunities to ensure that all FCPs can safely work toward MSK best practice standards in this environment [15]. The varied landscape requires new solutions to address the needs of FCPs and take into account the significant governance and role differences from a more traditional physiotherapy service model.

This research details the co-design of the Primary Care Individual Social Norms MSK Data Dashboard (PRISM) and guidance document. PRISM is a novel intervention that aims to address the need for real-world data feedback through targeted data visualization, enabling clinicians to reflect on their practice in FCP roles in line with local context filters and their knowledge of guidelines and quality indicators in primary care MSK [16-22]. PRISM includes a social norms feedback mechanism, a behavior change approach known to be effective in promoting behavior change, reducing unwarranted variation in clinical practice, and improving patient outcomes [23]. By showing clinicians summaries of their clinical behavior data relative to their peers, PRISM creates opportunities for reflection and supportive supervisory conversations. PRISM also includes a nudge mechanism where it shows collated MSK data for interventions that FCPs do not currently do but may consider. Interventions an individual has recorded rarely or not at all, such as smoking cessation, weight management, or health and well-being referrals, remain visible alongside those they use frequently. Finally, it includes context filters, such as body area or local demographics such as Index of Multiple Deprivation (IMD), age of population, and so forth, to help contextualize the data. The dashboard can be reviewed independently and in the learning environment of clinical supervision, “a professional development activity where the less experienced clinician can utilise the knowledge and experience of their supervisor, to address any gaps in knowledge or skill set and thereby improve their own clinical performance and patient quality of care” [24-26].

This work intends to anchor data-informed clinical reflection in the standards of evidence-based practice that define quality MSK care [27,28]. National Institute for Health and Care Excellence guidelines, clinical quality indicators, and best practice frameworks exist to guide clinical practice, but translating them into meaningful, real-world metrics for a relatively new and structurally complex role such as FCP is not straightforward. The FCP clinicians, service leads, and national leaders best placed to do this translation are those who have accumulated deep working knowledge of both the evidence base and the realities of FCP practice. They are, in effect, walking repositories of the National Institute for Health and Care Excellence guidance and MSK standards that a quality dashboard should reflect. Co-design with these experts is therefore the most direct route to ensuring that the metrics underpinning the PRISM dashboard are grounded in the evidence base for musculoskeletal care [29]. A 2026 evaluation of FCP service provision by Getting It Right First Time with National Health Service (NHS) England and the Chartered Society of Physiotherapy consolidated evidence for ongoing variation in FCP governance, supervision, and service configuration across England reinforcing the need for quality improvement tools of this kind and timeliness of this work [30].

Research question: Can we coproduce the PRISM dashboard and guidance document with experts to select metrics that best capture quality in MSK care?

- Objective 1: To co-design the metrics, filters, and guidance content of the PRISM dashboard and guidebook that best exemplified evidence-based practice and reflected the real-world clinical context of FCP roles in primary care.
- Objective 2: To apply a critical realist (CR) analytical approach to the data to identify the contextual mechanisms, barriers, and enablers that shaped how PRISM should be co-designed for primary care.

## Methods

### Design

#### Overview

This research is a qualitative co-design study using workshop methodology and thematic analysis through a CR approach [31]. This research used coproduction principles of “a shared understanding, identifying and meeting needs, giving everyone a voice and sense of ownership, and creating trust and confidence” [29]. All stakeholders had equal contributing power in the workshop environment [29,32]. Co-design was an important part of the coproduction process. It is commonly used in intervention design in primary care [33] and digital health [34,35].

This research used a series of workshops bringing together stakeholders in a PRISM dashboard think tank. It engaged clinical FCPs and nonclinical physiotherapy stakeholders, such as managers and national FCP leaders, as well as a patient representative who considered implications on evidenced and quality patient care. This process aimed to build trust and create ownership in the PRISM intervention. The group helped capture real-world needs and informed uptake and adoption for future stages of the PRISM lifecycle [34].

#### Objectives of Workshops

The objectives are as follows:

1. Workshop 1: Establish requirements of the PRISM dashboard, including:
  - Identifying user needs,
  - Identifying key metrics that characterize the clinical activity in FCP practice and mapping them to quality indicators in MSK care,
  - Identifying filters that would give more meaning to data comparisons, such as local IMD or age of population, and
  - Identifying barriers and facilitators
2. Workshop 2: Refine the characteristics of the PRISM dashboard and develop the PRISM guidance document, including:
  - Considering the experiences of individual FCPs and their supervisors using the dashboard,
  - Considering how to use the dashboard to nudge FCPs toward quality care, and
  - Considering what should be included in the guidebook.

### Participants and Recruitment

#### Overview

Recruitment took place over 3 months from February to March 2025. Participants were purposively sampled based on their professional standing or reputation as improvement-focused FCP leaders [36,37]. Participants were identified by the lead researcher (ED) through national and regional FCP clinical, research, and digital networks, and were invited by email with a participant information sheet. Ten of the 12 approached consented to take part and were sent an evidence summary and study rationale ahead of workshop 1 (Figure 1), and none withdrew. The participants often held more than 1 role. There were 10 participants in total. Seven of the participants were clinical physiotherapists in First Contact Physiotherapy across 5 different NHS trusts (Table 1).

Other roles were described in terms of their function to protect participants’ anonymity. One nonclinical physiotherapist was a national leader in physiotherapy digital innovation and data; 2 others held digital or data leadership roles locally. Two were national physiotherapy research leaders. Seven of the group were clinical and/or team leads in FCP. One person was a patient and public involvement representative who has been involved in this study since the funding application. Their role was to ensure that any impact of PRISM was considered in terms of the potential onward impact on patients.

**Figure 1.**
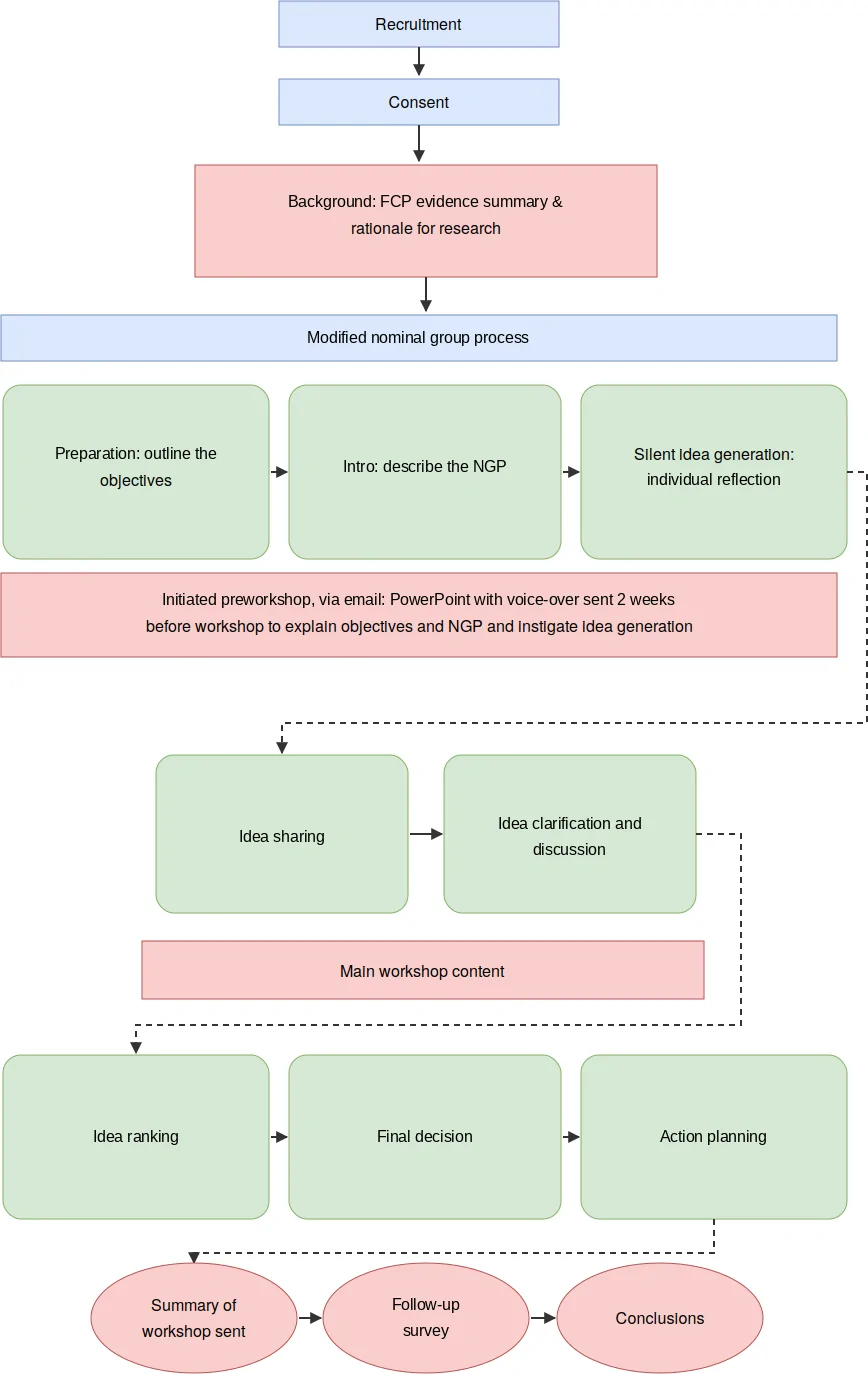
Procedure of modified nominal group process used to co-design the (Primary Care Individual Social Norms MSK Data Dashboard) PRISM dashboard and guidebook, showing preworkshop, workshop, and postworkshop stages across 2 online workshops held in June and August 2025, with 10 first contact practitioners and musculoskeletal stakeholders in National Health Service primary care in England. FCP: first contact practitioner; NGP: nominal group process.

**Table 1.** Roles held by the 10 participants in the PRISM^a^ co-design workshops. National Health Service primary care in England, June to August 2025. Roles are reported by function to protect anonymity, and participants often held more than 1 role.

Participant ID	FCP^b^	PPI^c^	Current or former clinical leader	Regional or national lead role	Data or information or digital physiotherapy leader	FCP supervisor
P01	Yes	No	Yes	Yes	No	Yes
P02	Yes	No	Yes	No	No	Yes
P03	Former	No	Yes	Yes	Yes	Former
P04	No	Yes	No	No	No	No
P05	Yes	No	Yes	Yes	No	Yes
P06	Yes	No	Yes	No	Yes	Former
P07	Yes	No	Yes	Yes	No	Yes
P08	No	No	No	Yes	No	No
P09	Yes	No	No	No	No	Yes
P10	Yes	No	Yes	No	Yes	No

a PRISM: Primary Care Individual Social Norms MSK Data Dashboard.

b FCP: first contact practitioner.

c PPI: patient and public involvement.

#### Inclusion and Exclusion Criteria

The inclusion and exclusion criteria are as follows (Textbox 1).

Textbox 1. **Inclusion and exclusion criteria.**

Inclusion criteria

- Worked as physiotherapy professional or lead work in primary care.
- Physiotherapy data knowledge and experience.
- Patient representative with knowledge of first contact practitioner (FCP) landscape.
- Available for 3 workshops of 90 minutes duration.
- English speaking.
- Able to use MS Teams for meeting purposes.

Exclusion criteria

- Unable to commit to the research.
- Lack of experience with FCP data.

### Procedure: Modified Nominal Group Process

The workshops were conducted using a modified nominal group process (NGP), a decision-making, consensus-building methodology where equal contributors aim to solve a problem. It gave every participant an equal voice in shaping the dashboard’s priorities through the use of the ranking and feedback surveys. The standard stages are as follows:

1. Preparation: outline the objectives.
2. Introduction: describe the NGP and establish rules.
3. Silent idea generation: individual reflection on objectives to rank and suggest talking points.
4. Idea sharing: share in turn. ED to facilitate.
5. Idea clarification and discussion: insights and clarifications.
6. Idea ranking: prioritization of ideas and identification of sticking points.
7. Final decision: synthesis and consensus are reached.
8. Action planning: next steps [36].

The workshop was modified to accommodate the shorter time that participants were able to give. For both workshops, steps 1‐3 were conducted over email, as were steps 6‐8 [38]. Preparation and idea generation were prompted through the use of a video introduction and preliminary survey based on existing evidence [15,39] sent before workshop 1. As per Figure 1, idea sharing and clarification, followed by group discussion, formed the bulk of the workshop. Following workshop 1, outputs were synthesized by the lead researcher (ED) into a structured summary of the metrics and quality indicators participants had ranked and discussed, alongside the 3 filter categories identified by participants (service, FCP, and patient-level filters) and a proposed threshold for defining variation. This summary was circulated to participants by email as per the postworkshop and preworkshop stages (Figure 1) and was presented back to the group as a recap at the start of workshop 2. The metrics were discussed again in workshop 2 as context; however, workshop 2 had different objectives. In line with guidance, training and support were offered to any participant or PPI that would like to engage in workshops or data analysis [32]. Workshop 1 was conducted on June 12, 2025 (1 PM to 3 PM), and workshop 2 was conducted on August 21, 2025 (11 AM to 1 PM).

### Ethical Considerations

Ethical approval was granted by the University College London Research Ethics Committee for Life and Medical Sciences (project ID 0399) on February 17, 2025. All participants gave informed consent, including consent to recording and to the use of anonymized quotations. Transcripts were pseudonymized, participants are identified by number only, and roles are reported by function to protect anonymity. A patient representative was involved throughout to ensure that any effect of PRISM on FCP behavior was considered in terms of its onward consequences for patients. No compensation was offered for participation

### Data Collection

Workshops were conducted online and were 90 minutes in length. The workshops were recorded in MS Teams. Breakout rooms were used to discuss key topic areas; these were also recorded and the whole group discussed the outputs of each breakout. Workshops were facilitated by ED. Workshop 1 was focused on parameters of the dashboard including identifying user needs, identifying key metrics that characterize the clinical activity in FCP practice, and identifying filters that could shed light on the variation in FCP data, such as local IMDs or age of population (Table S1 in Multimedia Appendix 1). Workshop 2 focused on the human factors, unintended consequences, and the content and use of the PRISM guidebook (Table S2 in Multimedia Appendix 1). Before the first workshop and following each workshop, participants were emailed to rank and comment on the evidence and data from the workshop toward conclusive findings. This was an important step to capture the unique perspective of each participant.

### Data Analysis

Data from each workshop were analyzed by the lead author (ED), a clinical academic physiotherapist in digital health. ED has experiential knowledge of the clinical and service context of FCP and may have shaped how this research has been developed and delivered and how the resulting data were interpreted. This knowledge was predominantly helpful, and potential areas of bias were managed by discussion with the wider team who were not FCPs [40]. In general, participants’ familiarity with FCP practice allowed discussion to move quickly to substantive issues without lengthy explanation of the role. While the NGP was designed to reach consensus on priority metrics for FCP practice, the expert participants generated considerably richer contextual data than metrics alone, including accounts of the structural conditions, governance pressures, and workforce factors that shape variation in FCP care. Critical realism states that observable events are produced by underlying generative mechanisms, which operate or lie dormant depending on the conditions in which they sit. This suited a study that set out to explain the conditions under which social norms feedback could change FCP practice. A CR approach was chosen with the aims to identify the causal structures that underpin real-world outcomes. It was particularly well suited to this research where understanding the participants’ context, the FCP landscape, and the conditions in which PRISM would have to be implemented in primary care were fundamental to understanding how the intervention might function.

The transcription of the workshops along with notes taken at the workshop was synthesized by the research team to rapidly inform next steps of the workshop process. A full analysis of the transcripts was completed following Fryer’s [41] CR approach to thematic analysis, using NVivo (version 12 Plus; QSR International) [42]. ED imported the data files from MS Teams into NVivo (version 12 Plus). The data were coded line by line, using inductive descriptive codes alongside a small number of a priori codes from the topic guides. Nodes were organized into hierarchical categories of child and parent nodes, with d included in multiple nodes [42]. Codes addressing the same themes were grouped into categories, and patterns or differences were noted, and competing perspectives coded and kept. Refined categories were reviewed by FS.

The consensus workshop process and CR thematic analysis operated as 2 parallel analytic strands applied to the same workshop data. The consensus process identified the specific metrics, quality indicators, and filters that populate the dashboard itself, while thematic analysis surfaced the governance and structural conditions shaping how those metrics would need to be used and interpreted in practice. The latter directly informed the design of the guidebook and the supervisory and reflective practice features of PRISM, ensuring that the dashboard’s content and its conditions of use were developed in parallel.

## Results

### Consensus Output: Co-Designing a Core Metric Set for FCP Practice

Despite significant service variation across sites, participants broadly agreed on a core set of metrics capable of describing clinical practice for FCPs (Textbox 2). They identified that while service variation will lead to inconsistencies, a core set of metrics would be likely in common across multiple sites. They identified that some metrics, such as referrals to secondary care, imaging, or prescribing, were pathway or skill-dependent metrics that were contingent on local diagnostic access, prescribing rights, and referral pathways. For example, some primary care areas or employers do not allow FCPs to request blood tests or magnetic resonance imaging without a sign-off by a GP.

Textbox 2. **The final list of metrics. Core: applicable across the majority of first contact practitioner services regardless of local configuration. Pathway-dependent: contingent on local service configuration, diagnostic access, or prescribing rights or skills. Agreed across 2 online co-design workshops with 10 musculoskeletal stakeholders, National Health Service primary care in England, June to August 2025.**

Clinical activity

- Core: exercise prescription, self-management or condition advice, shared decision-making tool use, personalized care plan completion, and risk stratification tool use (eg, STarT Back)

Diagnostics and investigations

- Core: refer to general practitioner (GP) for diagnostics or imaging
- Pathway-dependent: magnetic resonance imaging request, X-ray request, computed tomographic scan request, ultrasound request, dual-energy X-ray absorptiometry scan request, nerve conduction study, and blood test request

Prescribing and procedural support

- Core: over-the-counter medication advice, refer to GP for medication or injection
- Pathway-dependent: opioid prescribing, nonsteroidal anti-inflammatory drug prescribing (topical and oral), neuropathic pain medication, antidepressant prescribing, bisphosphonate prescribing, deprescribing, and first contact practitioner (FCP) injection

Community therapies and Additional Roles Reimbursement Scheme referrals

- Core: physiotherapy referral, community rehabilitation physio, occupational therapy, podiatry, dietician, community pain service, improving access to psychological therapies or mental health professional, pharmacy, nurse, paramedic, community exercise scheme, falls or frailty referral, single point of access, Musculoskeletal Clinical Assessment and Triage or musculoskeletal specialist, and interface services

Personalized care referrals

- Core: social prescribing, health and well-being practitioner, employment support services, National Diabetes Prevention Programme, smoking cessation, weight management services, and FitNote (personalized care)

Medical escalation and primary care activity

- Core: refer to GP for medical escalation, refer to GP for onward referral, FCP FitNote, indirect GP support: safeguarding, and indirect GP support: complex case review

Secondary care referrals

- Core: accident and emergency (A&E) and urgent A&E
- Pathway-dependent: orthopedics (routine and urgent), neurology (routine and urgent), rheumatology (routine and urgent), advice and guidance, secondary care pain service, and women's health or pelvic pain

Participants were frustrated that metrics may struggle to best capture a well-rounded clinical conversation. The inability to capture informal clinical activity such as practicing exercises or detailed conversations about health beliefs was lamented but was felt to be beyond data characterization. Instead, participants agreed that the presence of health referrals as well as social and psychological referrals over time indicated that biopsychosocial conversations were happening, making referral patterns a meaningful proxy for the breadth of clinical practice. The group also felt that the list of metrics would need to be iterative and adaptable as FCP services evolve.

### Identifying Filters That Would Give More Meaning to Data Comparisons

Contextual filters were identified as essential to giving the metric data meaning. Participants agreed that raw activity rates are difficult to interpret without understanding the population served, the clinician’s experience, and the service configuration in which they work. Three categories of filter were co-designed: patient-level, clinician-level, and service-level (Textbox 3). Population demographics filters were seen as particularly important for capturing caseload complexity and supporting a knowledge of population health. The group discussed how population demographics filters may go some way to capturing caseload complexity at the practice where FCPs work (Textbox 3).

*Major indicators of ill health, you know, basically housing problems and school problems and, you know, legal problems and all these kind of things...That loneliness, isolation, all the type of things that are real contributors to health*.

[P03]

The group also discussed filters that pertain to clinical experience and service-level filters. They are not available in PRISM but are collected as part of related feasibility trial [43].

Textbox 3. **Contextual filters at patient, clinician, and service levels, co-designed to support interpretation of first contact practitioner activity metrics, from 2 online workshops with 10 musculoskeletal stakeholders, National Health Service primary care in England, June to August 2025.**

Patient-level filters

- Demographics: age, ethnicity, BMI, employment status, and physical activity level
- Clinical: body site, frequent flyer status (≥3 consultations in 6 months), comorbidities and long-term conditions, and Index of Multiple Deprivation

Clinician-level filters

- Background and role: years qualified, education level (BSc, MSc, etc), band, time working as a first contact practitioner, full-time equivalent percentage, and appointment length
- Skills and support: access to supervision, access to training and development, advanced practice skills, injector, and prescriber

Service-level filters

- Configuration: local pathways, referral rights, diagnostic access, prescribing access, available local services, and new patient: follow-up ratio

### Theme 1: PRISM as a Tool for Clinical Learning and Reflective Practice

Participants discussed the potential of PRISM across a range of functions that could offer something novel and helpful to clinical learning. Supervisors and FCPs agreed that there is a particular challenge in meeting in person in the clinical environment which might allow the supervisor to witness and better understand the FCP’s practice. This type of shadowing in supervision would be typical in a community services environment. Participants saw in PRISM the potential to transform FCP supervisory conversations by presenting a version of shadowing by creating the opportunity to ground supervision in real-world data. Rather than relying on the FCP to identify their own learning needs, PRISM provides opportunities to reflect on actual clinical activity patterns. Supervisors and team leads considered that PRISM may “‘give me a flag’ that would steer the direction of supervision” [P01].

*At present supervision isn’t necessarily data driven...this would then significantly help the supervisor and the supervisee to have something in numbers to see what’s happening and to trigger discussion about those particular cases*.

[P10]

The potential to bring deeper understanding to clinicians and supervisors about the flow of MSK patients from primary care into other areas was highlighted. Both FCPs and supervisors felt that they had assumptions about where patients go, and they welcomed the opportunity to reflect on real data.

*It’s a reflective type thing for me...sometimes I feel like I’ve referred everybody, and then I look and actually it’s only a couple. The data balances it out*.

[P02]

*It helps identify patterns we’ve historically had blind spots to, like vocational issues*.

[P08]

A broader opportunity identified by participants was PRISM’s potential to support FCPs in embracing the full scope of their primary care role. Patients who see an FCP rather than their GP may not have another health care contact for some time, making each FCP encounter an opportunity to identify and act on wider health needs beyond the presenting MSK complaint. This aligns with the NHS Making Every Contact Count framework [44], which places prevention and health behavior change at the heart of every primary care contact. Participants recognized that FCPs have historically been trained as MSK specialists, and that the transition to primary care requires a conscious broadening of clinical focus to include public health priorities such as smoking cessation, weight management, and the wider determinants of health relevant to Core20PLUS5 populations [45].

*We’re very good at MSK—but what are we missing about public health, social care, wider determinants when patients see us instead of a GP?*

[P03]

*Linking in with some of the national things, if we think about like smoking cessation, obesity, weight management services, these things are really important things nationally and those are things we probably really want to change behaviour on*.

[P06]

*Showing that people aren’t coding or using social prescribing will nudge people to do it*.

[P08]

PRISM’s nudge mechanism directly supports this by surfacing data on interventions that FCPs are not currently using but could consider. It prompts clinicians to look beyond the MSK consultation and act on the wider health needs of their patients. With high levels of stress and burnout identified in the FCP cohort, participants identified that the data and supervisory conversation also provide an important opportunity to reassure and affirm good practice. Regardless of whether FCPs are outliers or within norms, context filters and supervisory conversations can reaffirm that practice is safe and appropriate.

*There is a bit of reassurance in the data sometimes...seeing yourself sitting within the norms, thinking “OK, I am doing an all right job here despite the challenges*.”

[P02]

*If you feel you’re in the 80%, that’s empowering...You’ve got some wiggle room and can feel confident about your practice*.

[P08]

FCPs also identified an openness to being nudged toward interventions they don’t usually consider, recognizing that they cannot always meet all of their patients’ needs alone.

*It means that I’m sort of thinking that, OK, I can't meet all my patient’s needs, but what else could I send them?*

[P02]

Although PRISM is an individual tool, participants recognized that it creates insights clinicians can choose to share with colleagues, enabling a team-level learning culture that extends beyond the individual supervisory relationship.

*That shared learning could be a big benefit*.

[P07]

*You can use people who are doing more of something well to share how they do it with others*.

[P02]

### Theme 2: Structural Variation, Role Complexity, and the Conditions for Safe Implementation of PRISM

FCPs and supervisors identified a paradigm shift between a traditional physiotherapy role and working as an FCP. The variation between primary care practitioners, where diagnostics and MSK triage may be more important than traditional rehabilitation skills, leaves some clinicians unsure of the scope of their role and whether they are performing well. Participants felt that PRISM presents an opportunity to support clinicians to explore their activity across the full breadth of the FCP role.

FCPs themselves vary significantly in experience, skill sets, education, and knowledge. The governance of services was thought to vary considerably in pathways, integration into primary care teams, levels of supervision, support, training, and access to diagnostic resources. Participants suggested that this variation may represent causal conditions under which unwarranted variation in FCP practice arises.

*There’s undoubted variation across services*.

[P01]

*There’s going to be huge variation in service delivery...so it’s how we capture that*.

[P06]

*Even between surgeries, someone had like a very different format and a very different set of outcomes to what others would let you do, so there was variation even within me working in one practice versus another practice*.

[P03]

*I always find it really interesting—as an FCP I could do a CT scan in one clinic. And we could do an MRI in the (M)CATs clinic and I couldn’t do an MRI in the other clinic. It’s like...it’s crazy, isn’t it?*

[P03]

Participants were also alert to the ways in which this structural vulnerability shapes how PRISM itself might be experienced. With high levels of stress and burnout already identified in the FCP cohort, the concern was raised that social norms feedback could land very differently depending on the quality of governance and supervisory support surrounding the individual clinician. Where supervision is inconsistent, where FCPs feel isolated or scrutinized, and where the organizational culture is not psychologically safe, data showing an FCP as an outlier risk being experienced as judgment instead of an invitation to reflect.

*I just thought any individual was going to say, well you’re ranking me. So that’s definitely performance pressure*.

[P04]

For this reason, PRISM needs to be openly discussed as a nonjudgmental tool for reflective practice in order for its safe use across teams with varied governance. The concern in this case was with the conditions under which the data are received. Data and social norms are neither good nor bad in themselves; they must be understood in local context, with consideration of the clinician’s scope of practice and complexity of caseload.

*It opens discussion rather than being persecutory—it’s a discussion point*.

[P07]

In the supervisory context, local pathways and service configuration are well understood, making supervisors well placed to support individual FCPs to interpret their data in the context of their peers and their local practice. Participants emphasized that meaningful and safe use of PRISM in supervision will always depend on that contextualized conversation. As one participant put it simply: “it’s all going to depend on that conversation” [P07]. The design of PRISM to some degree assumes that clinical supervision is available and a healthy learning environment; however, the tool is also designed to work in reflective practice for an individual, allowing them to consider the variation in their practice but perhaps with fewer levers for change than those who use it in supervision.

### Theme 3: Data Integrity and Implementation Risk

A fundamental challenge for any FCP primary care data tool is the near-impossibility of extracting reliable service-level data directly from electronic patient records. The group acknowledged that extracting service-level data from primary care often had insurmountable operational barriers, with years of effort yielding little progress.

*They go into a black hole and there’s nothing ever, never comes out*.

[P07]

*Working with [colleagues] for the last 4‐5 years trying to answer the data access question*.

[P05]

PRISM therefore relies on a self-populating data collection form completed by FCPs, which offers real advantages; clinical leads have their data at their fingertips and can respond to service questions by interrogating it. However, it introduces 3 implementation risks that participants identified and interrogated carefully.

The first is reliability. Asking clinicians to tick a box to record a clinical activity is inherently less reliable than automated extraction and introduces the need for ongoing data audit. Participants grappled with the particular challenge of capturing complex clinical activities, such as shared decision-making, through simple data fields.

*Shared decision making...this is too subjective, leaves no audit trail and seems unreliable. On the other hand, a box tick that says shared decision making tool used, leaves an audit trail where the tool could be identified in the record*.

[P08]

The second is data gaming. Where social norms feedback shows a clinician is an outlier, there is a risk they respond by inflating their data recording rather than changing their practice.

*If as a clinician you got feedback that you weren’t providing as many personalised care plans, you would then probably just start thinking everything you’d give as a personalised care plan. And therefore there’s probably no quality control about what is and isn’t, and therefore that data’s probably not gonna be that useful*.

[P06]

The third is clinician burden. The group weighed the value of granular data against the cost of collecting it, recognizing that time is a scarce resource in FCP clinics.

*It’s a waste of time and we know that time is massively precious within these clinics*.

[P08]

*It’s not easy...one that goes into taking extra time. But then we have the absolute full granularity in the ability to export and interrogate*.

[P03]

Participants accepted these tensions, concluding that careful metric selection, clear data definitions, and regular audit represent the most pragmatic approach to managing data quality in this setting. As one participant summarized: “It’s probably our best guess, right?” [P06]

### Theme 4: Co-Designing the PRISM Guidebook as a Knowledge Support System for FCPs

Across both workshops, participants consistently identified that the metrics and dashboard alone would be insufficient without a structured knowledge support system to guide how PRISM is used in practice. The guidebook was identified as an essential component of the intervention both as a practical means of learning to understand data visualizations and storytelling and as a place to outline the principles of safe, contextualized, and reflective use of PRISM for both FCPs and their supervisors.

The central principle participants agreed on was that any behavior change arising from PRISM should come through a combination of behavior change mechanisms triggering actionable insights and impetus to change, blended with informed reflective practice, clinical knowledge, and local context. The guidebook must communicate clearly that data and social norms are open to interpretation, and that the dashboard is a starting point for conversation. Participants strongly felt that video examples of how to use the data in a contextualized, nonjudgmental way would be particularly valuable.

Participants also identified the need for distinct guidance for the 2 user groups, FCPs and clinical supervisors, recognizing that each brings a different perspective to the data and requires different support to use it well. The group co-designed a structured 4-step reflection approach for use in supervision: Describe, Interpret, Contextualize, and Plan. This provided a practical framework for turning data into learning.

The co-designed content priorities for the guidebook are summarized in Table 2, organized across 6 sections covering how PRISM works, who it is for, how to access it, how to approach the data, how to interpret the visualizations, and how to connect metrics to evidence-based practice and quality care.

**Table 2.** Content priorities for the Primary Care Individual Social Norms MSK Data Dashboard (PRISM) guidebook, agreed by consensus across 2 online co-design workshops with 10 musculoskeletal stakeholders, National Health Service primary care in England, June to August 2025. Data were organized into 6 sections covering how PRISM works, who it is for, how to access it, how to approach the data, how to interpret the visualizations, and how metrics connect to evidence-based practice.

Section	Key content
1. How PRISM works	Explains the mechanisms: social norms feedback (primary), nudging (secondary), and reflective practice both as an individual and in supervision. Explains data groupings: the individual, provider peers, and all FCPs^a^ in the data. Emphasizes that change arises through reflection and professional judgment.
2. Who PRISM is for	Distinct guidance for 2 user groups: FCPs and clinical supervisors or FCP leads.
3. Accessing PRISM	Step-by-step login guidance for Tableau Cloud with 2-factor authentication.
4. Structured approach to the data	4-step reflection structure: Describe, Interpret, Contextualize, and Plan. Explore patterns starting in outcome box, use context filters, and identify learning. Supervisor tasks: contextualize data and reinforce nonjudgmental interpretation.
5. Understanding the visualizations	Describes each graph or chart and what it shows. Explains the use of centiles. Explains 3 comparison groups: the individual, provider peers, and all FCPs.
6. Use cases that anchor the metrics in quality care and evidence-based practice	Highlights evidence-based use cases of PRISM that help users to understand their data in the context of national evidence and quality benchmarks.

a FCPs: first contact practitioners.

## Discussion

### Principal Findings

The key findings of this paper were that expert participants reached consensus on a core metric set describing the breadth of the FCP role, together with patient, clinician, and service-level filters. The CR analysis added important contextual considerations where variations in structure and governance are known to exist and demonstrate challenges for equitable experience of PRISM. National evaluation has established that FCP services deliver clinical and cost-effective MSK care at service level [5,6]. However, the consistency with which that care is applied by individuals has been questioned. A National Health Service—England–led national FCP evaluation with 229 responses covering approximately 16% of Primary Care Networks in England found substantial unwarranted variation across FCP service delivery and identified data collection, alongside workforce assurance, scope of practice, and governance, as underlying structural causes of that variation which prevent the benefits of the role being realized [30]. While 61.6% of services reported collecting performance and quality metrics, the evaluation could not establish which metrics these were, how often they were collected, or what effect they had. Two of the report’s concluding “enablers of good practice” address this directly, calling for FCP services to routinely collect, share, and analyze performance and quality metrics in order to embed a learning health system approach [46,47].

Through a CR lens, the semipredictable patterns of governance variation, training, and supervision variation identified in the evaluation were explored in this research as a way of understanding design requirements. Retroduction, which means asking what conditions would have to be in place for the observed pattern to occur, moved the analysis from the variation participants described to the structures that generate it, including prescribing rights, diagnostic access, referral pathways, and the availability of supervision. Further hidden structures may include levels of support, risk tolerance, and the cultural shift to work in primary care. These arrangements are not visible in activity data, but they determine what an individual clinician is able to do and how feedback on that activity is received, so they had to be accommodated in the PRISM design. The guidebook outlines these findings clearly, where the narrative supports users in relation to nonjudgmental data interpretation, reflective practice, use cases that touch on hidden mechanisms and the accommodation of service variation in the use model. PRISM co-design moves from the position that we should collect data to the conditions under which that recommendation produces learning and actionable insights.

Despite significant variation in FCP services across sites, participants reached consensus on a core and variable metric set capable of describing clinical activity across the breadth of the FCP role. They also separated variation generated by clinical decision-making from variation generated by the structures within which those decisions are made, such as local demographics, pathway factors including diagnostic access, and prescribing authority. In order for PRISM to exist in this context, participants designed an environment where PRISM presents metrics with local “context filters” built in for users to contextualize the data by IMD or age of population, and so forth. [29]. The contextual filters can inform population health and inequalities that can impact clinical practice [45]. This is consistent with comparable dashboard co-design studies, where building in contextual depth to explore local variation is a recognized feature of successful implementation [48,49]. Ross et al [50] identified flexibility to local context, respect for limited clinician resources, and genuine attention to end user needs as critical conditions for implementing digital health in NHS primary care, all of which are embedded in PRISM’s co-design through its metric structure, contextual filters, and guidebook framework.

Participants were clear that the metric set describes what was done in a consultation and cannot describe how well it was done. The elements they most regretted losing, such as coaching a patient through an exercise or working through health beliefs in conversation, are the elements that build the therapeutic relationship and carry person-centered care. The therapeutic relationship is associated with patient satisfaction and adherence to treatment in rehabilitation populations [51], and with health care outcomes more broadly [52]. Their absence from the dataset reflects the limits of what can be recorded. Participants judged that referral patterns over time act as a reasonable proxy for the breadth of practice, but the guidebook is clear that this is a starting point for conversation and reflection where other unmeasurable factors should also be considered.

The role of supervisors in supporting actionable insights from the PRISM dashboard is central to one of the key conflicts of how acceptable PRISM is to FCP users, and whether it is perceived as a positive reflection tool or whether it is used negatively as a performance management tool. Clinical supervision is associated with improvements in organizational outcomes, patient safety, and effectiveness of care [24-26]. However, nationally, 12.2% of FCPs have no allocated clinical or CPD supervision time, and a further 16.6% of services do not know whether it is included in the job plan [30]. This matters because supervision quality depends less on individual supervisor skill than on how well the FCP role is understood by the organization around it [53]. Participants reported that where supervision, role clarity, and psychological safety are present, comparison with peers can generate reflection. Where they are absent, the same comparison can generate defensiveness or a perception of surveillance. The results have shown that the experience of using PRISM would be shaped by the contextual factors around the data. An important finding for PRISM is that a tool that depended on supervision would work least well in the services with the weakest structural support, which are the same services in which unwarranted variation is most likely to arise. Although PRISM is designed to be used in supervision, which is consistent with evidence that feedback is more effective when delivered by a trusted source alongside explicit goals and action planning [54], the option for stand-alone reflective use is therefore an essential design requirement.

For social norms, medians and interquartile ranges were set in order to define variation, which is consistent with previous research [23,55]. Participants were clear that norms are a useful trigger for critical reflection rather than an indicator of quality in themselves. Two functions of the comparison emerged that receive less attention in the social norms literature. The first is affirmation. Given the levels of stress and burnout reported in this workforce, participants valued the prospect of seeing their practice sit within the norms as evidence of working safely under difficult conditions. The second is scope, since participants saw interventions they do not currently use displayed alongside their own activity as a prompt to act on the wider health needs of patients who may have no other support from primary care. The wider social norms literature identifies the risk of “norm-chasing” behavior, where clinicians shift toward perceived norms without clinical justification; for example, if norms show a normative range for number of referrals to orthopedics, then clinicians may aim to be inside the norms, whether or not it is appropriate for them. The PRISM guidebook and training are designed specifically to address this by positioning the dashboard as a starting point for structured reflection rather than an indication of good or bad practice; this includes analyzing whether it is good or bad to be in the “norms.” The guidebook sets out use cases that aim to connect metrics to the relevant MSK guidance and to broader primary care priorities including Making Every Contact Count and Core20PLUS5 [17-20,44,45,56,57], so that the interventions surfaced by the norms can be weighed against the evidence for them. A forthcoming paper mapping PRISM’s metrics against the National MSK guidance and quality indicators for MSK in primary care will add to this discussion.

Data quality was evident as a crosscutting implementation concern that is particularly acute in primary care, where extracting service-level data directly from electronic patient records remains practically impossible for most FCP services. Participants described sometimes years of attempting to access data across their primary care structure. This is a semipredictable pattern, holding across multiple services and clinical systems, and despite sustained effort by people well placed to succeed. Reasoning back from it, the barrier is sometimes technical, where FCPs cannot be identified in data extraction due to heterogeneity in clinic set up on electronic patient record. Where the activity is recorded well, the barriers are systemic. FCP was rolled out and commissioned without the data infrastructure that would allow the service to evaluate itself and learn. The enablers set out in the national evaluation amount to a request to retrofit it to solve this issue. The self-populating form follows from that constraint and it carries an advantage participants valued, since clinical leads hold their own data and can interrogate it directly. The self-populating data is a pragmatic solution but introduces risks of reliability and clinician burden that careful metric selection, clear data definitions, and regular audit can manage but not eliminate. Data gaming is a further risk, and one activated by the feedback itself rather than by the collection method, which is why the framing set out in the guidebook that encourages clinician and supervisors to avoid performance management thinking is important to data quality. Concerns about data trust and reliability in dashboard environments are well documented in the literature [58,59] where participants reported that data they did not trust undermined engagement with the tool entirely. This reinforces the importance of the data audit as a quality mitigation built into PRISM’s co-design and the importance of establishing electronic patient record data pipelines in future.

The national policy context forms part of the structural conditions in which PRISM is being designed. In 2025, National Health Service—England accepted the recommendation of the Leng review [60] that physician associates should not see patients who have not already been seen by a GP. The physician associate role had similarities to FCP, yet the FCP role involves assessing undifferentiated MSK presentations without prior medical screening. For FCPs, the refreshed multiprofessional framework sets out the clinical capabilities expected at that level and provided an evidence base for FCPs to rely on in demonstrating competency [9]. However, ongoing practice review and data are needed. A service that was established at pace without the infrastructure to describe its own activity is poorly placed to answer questions about its scope when they arrive, and those questions are now being asked. Participants described the same uncertainty with clinicians unsure of what the role includes and whether they are performing it well. PRISM provides an opportunity to add to the evidence base for FCP and increase clinical learning and quality. PRISM’s capacity to provide transparent, auditable data on FCP clinical decision-making positions it as both a tool for individual reflective practice and a contribution to the professional evidence base for the FCP role during a period of NHS workforce restructuring.

### Strengths and Limitations

The principal strength of this study is the quality of the participant sample, whose collective depth of knowledge makes the metric set relevant and knowingly agile to changes in the field. The findings on governance variation and supervisory quality as critical factors in PRISM’s implementation risk and acceptability require further testing in an upcoming feasibility trial [43]. A CR analysis identifies mechanisms as tendencies, which is why the feasibility trial is required to establish whether the mechanisms identified here operate as anticipated.

The self-populating data collection approach introduces risks of recording reliability and potential data gaming. This can be somewhat mitigated by careful metric selection, clear definitions, and regular audit. This is an important area for improvement in future iterations of PRISM. Participants set a limit on what any metric set can achieve, judging that some of the most valuable parts of an FCP consultation cannot be captured in a data field, but capturing clinical activity over time was deemed a suitable proxy.

The participant sample does not include perspectives from FCP education providers or higher education institutions, nor from NHS England training hubs, which act as an interface between national policy and frontline FCP education. The study did not include representation from underserved or rural FCP services. The lead researcher (ED) both facilitated the workshops and led the analysis; reflexive practice and coauthor discussion were used to manage but not eliminate the potential for facilitation bias.

### Conclusions

PRISM is a novel, co-designed, evidence-based dashboard for MSK care. Expert co-design has produced a metric set that describes the breadth of the FCP role while accommodating the variation between services. The CR analysis identifies the structural conditions under which PRISM is likely to function as intended. Three key co-design conditions were identified that can carry directly into implementation; feedback can be understood with local pathway and population context alongside the clinician data to add meaning. Supervision improves how the data are used but cannot be assumed, so stand-alone reflective use has also to be supported. Data collected by clinicians themselves need clear definitions and regular audit if they are to be trusted. This work aligns well with the national recommendation from Getting It Right First Time that FCP services should collect performance and quality metrics. PRISM goes further toward a specification of which metrics, and the conditions in which they can produce learning.

## Supplementary material

10.2196/105148 Multimedia Appendix 1 Topic guide.

## Abbreviations

CR: critical realist
FCP: first contact practitioner/physiotherapist
GP: general practitioner
IMD: Index of Multiple Deprivation
MSK: musculoskeletal
NGP: nominal group process
NHS: National Health Service
PRISM: Primary Care Individual Social Norms MSK Data Dashboard
