# Supplementary material for: Co-Designing the Primary Care Individual Social Norms Musculoskeletal Data Dashboard (PRISM) for First Contact Physiotherapy: Consensus-Based Qualitative Study
Source: JMIR Form Res. 2026 Oct 20;10:e105148. doi: 10.2196/105148 (PMC13683990; doi:10.2196/105148)
Supplement: Supplementary file 1 [file formative-v10-e105148-s001.docx]

Multimedia Appendix 1

Table S1. Topic guide workshop 1.

| 1. Topic Guide - Establishing Requirements of the PRISM dashboard | 1. Who are the users and stakeholders? 2. What are the goals of the users and stakeholders    - Consider personal development    - Identifying blind spots    - Reducing unwarranted variation    - Cost efficient services 3. Which data points should we look at for social norms and nudging?    - Consider the Primary Care Quality Safety and Effectiveness metrics.    - Consider personalised care, community services, primary care and secondary care    - Diagnostics and physio based activity    - Which metrics are most well linked to evidence for quality care?    - Are patient rated outcome measures (PROMS)and patient rated experience measures (PREMS) valuable? 4. What contextual data filters will be important to exploring variation?  - Consider patient filters such as body area, age, IMD, employment. Consider physio filters such as experience, skill set, WTE. Service filters such as duration of appointment, referral rights etc  5. Consider what the ‘thresholds ’ should be. ie for GP antibiotic stewardship, the threshold was set at 20% . ‘ie you are prescribing at a higher rate than 80% of practices’. |
| --- | --- |

Table S2. Topic guide workshop 2.

| 2. Topic Guide - Refining characteristics of the PRISM dashboard and developing the PRISM Guidance Document  - To consider the experiences of individual FCPs and their supervisors using the dashboard - To consider how to use the dashboard to nudge FCPs toward quality care. - To consider what should be included in the guidebook | 1. What are the human factors to consider when using the dashboard to support reflective learning?  - What might change for the FCP experience of supervision? - What might change for the supervisor experience of supervision?  2. What might the unintended consequences be ?  - Prioritise clinical judgement over social norms - Data quality - Context of local population and service - Non judgemental - Confidential - Supportive  3. How do we safe guard against unintended consequences in the guidebook? 4. How do we make connections between metrics, evidence and quality care. 5. What guidance do we offer for interpreting the data? 6. How might the data be limited? |
| --- | --- |
